# Interactive, case-based seminars in the digitized pediatrics block internship from the students’ perspective

**DOI:** 10.3205/zma001420

**Published:** 2021-01-28

**Authors:** Andrea Heinzmann, Sebastian Bode, Johannes Forster, Jan Berger

**Affiliations:** 1 Universitätsklinikum Freiburg, Albert-Ludwigs-Universität, Zentrum für Kinder- und Jugendmedizin, Klinik für Allgemeine Kinder- und Jugendmedizin, Freiburg, Germany

**Keywords:** pediatrics, interactive learning units, formative questions, evaluation

## Abstract

We report on the pediatric block practice digitized due to the COVID-19 pandemic. Nineteen seminars were recorded, which represent the broad spectrum of pediatrics as comprehensively as possible, and made available on the learning platform ILIAS in a learning sequence. In order to increase attention and learning success formative questions were interspersed into the seminars. The evaluation of the students showed a high acceptance of this course. Especially the formative questions and the high time flexibility were appreciated. A major point of criticism was that not all questions were resolved immediately afterwards. The resolutions were inserted in the meantime. Parts of the digitized block practical course should therefore be used in the pediatric block practical course after the end of the corona restrictions after appropriate revision.

## Zusammenfassung

Wir berichten über das aufgrund der COVID-19-Pandemie digitalisierte pädiatrische Blockpraktikum. Es wurden neunzehn Seminare aufgezeichnet, die das weite Spektrum der Pädiatrie möglichst umfassend abbilden, und auf der Lernplattform ILIAS in einer Lernsequenz zur Verfügung gestellt. Zur Erhöhung der Aufmerksamkeit und des Lernerfolgs wurden formative Fragen in die Seminare eingestreut. Die Evaluation der Studierenden ergab eine hohe Akzeptanz dieser Lehrveranstaltung. Geschätzt wurden insbesondere die formativen Fragen und die hohe zeitliche Flexibilität. Ein wesentlicher Kritikpunkt war, dass nicht alle Fragen im direkten Anschluss aufgelöst wurden. Die Auflösungen wurden zwischenzeitlich eingefügt. Teile des digitalisierten Blockpraktikums sollen nach entsprechender Überarbeitung daher auch nach Ende der Corona-Beschränkungen im pädiatrischen Blockpraktikum genutzt werden.

## Introduction

In the previous two-week block internship in paediatrics, the students examined patients daily under the supervision of a medical tutor. This is supplemented by a daily debriefing with the tutor, a two-hour observation in a pediatric practice and twelve one-hour seminars. Students also have access to an ELearning unit (pediatric examination methods and standard procedures) on ILIAS, the learning platform of the University of Freiburg.

Due to the COVID-19 pandemic a digital course had to be created within a very short time as a complete replacement for the block practical course pediatrics. At the time of planning and implementation, attendance courses and thus bedside teaching were prohibited. In addition, the University of Freiburg recommended asynchronous courses because of concerns about overloading the ILIAS server capacities.

## Project description

The twelve seminars that have been held so far have been digitized as interactive learning units (videos). For this purpose, the lecturers received a one-page summary of the most important recommendations for the creation of instructional videos [1], [2]. This included, for example, the request to record short video segments with a maximum length of 5-15 minutes, as well as the advice not to speak too clearly and deliberately slowly. The most important aspect was the request to ask students questions directly in the seminar, as this repeated testing has been shown to increase the attention and learning success of off- and online courses [3], [4], [5]. There was no specification of the question type. Multiple-choice (MC) questions, which had to be answered by the students with a click, as well as open-ended questions, which were to be worked on with a corresponding free text commentary, were possible. The MC questions were solved directly afterwards. 

In addition, the lecturers were filmed during the audio recording of the seminar in an informal setting and then made visible to the students as "talking heads" in the digitized seminars. 

**Figure 1:**
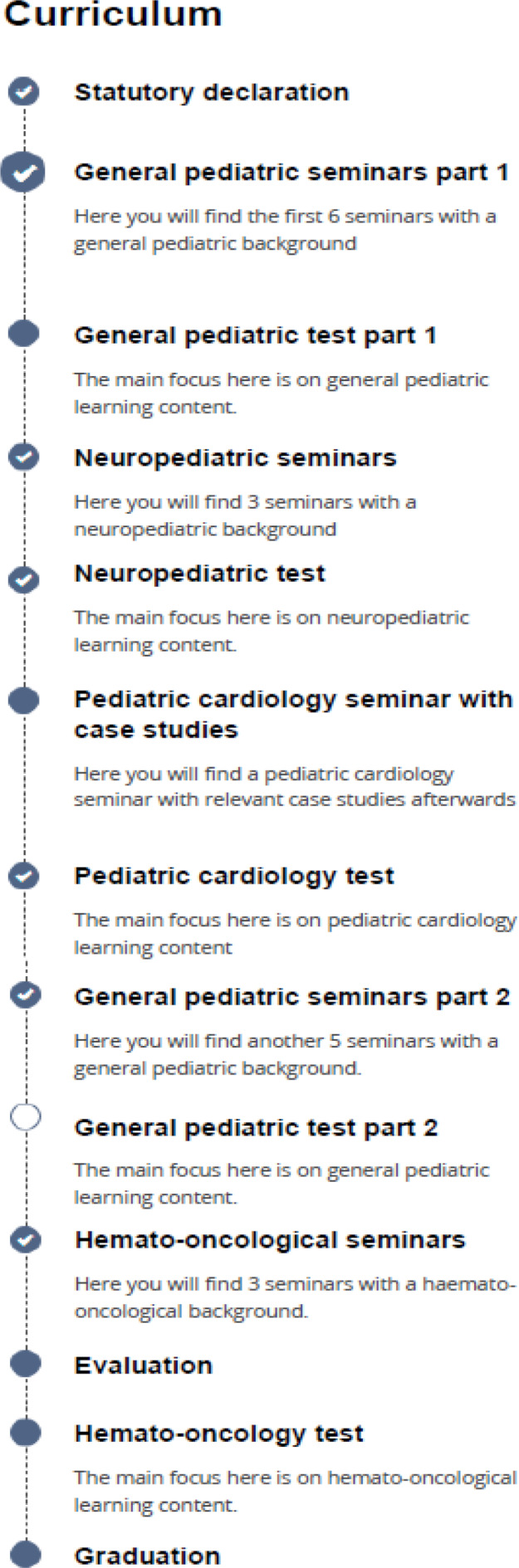
Complete structure of the pediatric block course on ILIAS. Empty circle: not yet processed. Blue circle: processing started. Circle with a tick: processing completed.

In addition, seven new seminars were developed and also digitized to supplement the teaching content that had previously only been taught in practical classes, for example on the normal neurological development of the child and a U9 in a pediatrician's practice (see attachment 1 for a list of all seminars). The seminars were interrupted by formative questions in order to increase the learning effect and attention [6], [7]. A total of 110 MC and 57 free text questions were used, so that an average of nine questions per seminar were used, in accordance with the recommendations of a study by Cook et al. [8]. The seminars, each lasting about 45 minutes, were divided into five thematic blocks and presented in a clear learning sequence on ILIAS (see figure 1 (Fig. 1)). Each block was followed by a summary learning control using MC questions. The evaluation was done via EvaSys^©^. There were 8 open questions with free text answers and 13 questions with a six-level Lickert scale (fully applicable to not applicable).

## Results

150 students – 78% of those originally registered – took part in the course and evaluated it. The digital block internship was evaluated with a mean value (mw) of 1.9 and a median (md) of 2.0, in comparison, the original format was evaluated with an average of 1.7. The students stated that they had learned a lot in the course (mw=2.1, md=2.0) and that they had regularly prepared and followed up (mw=2.5, md=2.0). The working conditions at home were very good for most students with mw=1.8 (md=1.0). The question of whether the changed learning situation was very stressful was answered in the negative with 4.3 (md=5). Only 6% had organized group work with fellow students; 42% wanted to do so.

**Figure 2:**
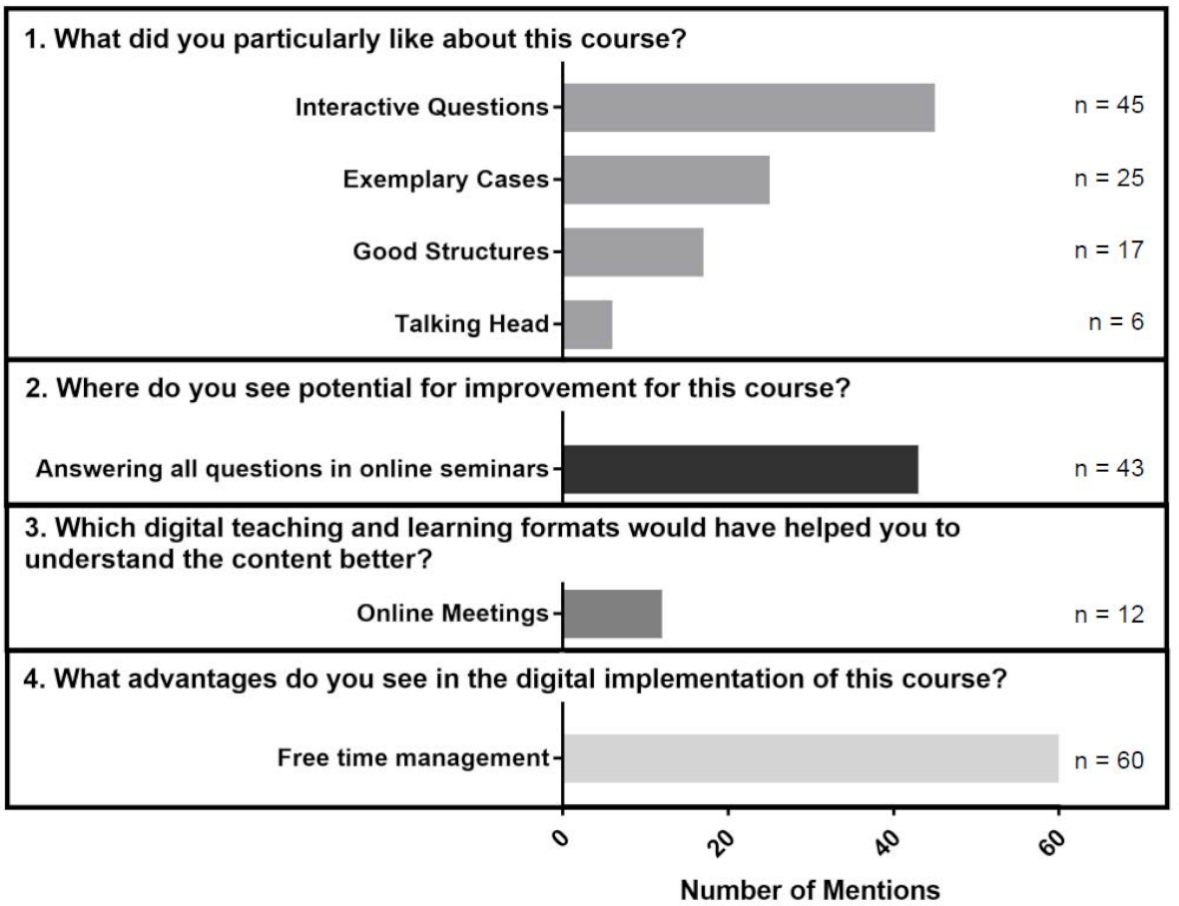
Number of mentions in free text comments. Multiple answers were possible.

In the free text comments, 25 students indicated that the questions they asked increased their attention and involvement with the course material. Further results of the free text comments are summarized in figure 2 (Fig. 2). 

## Discussion

In the student evaluation, the interactive questions in particular were positively emphasized, which subjectively increased attention and the learning effect. Roediger et al. in particular were able to show in several studies that (repetitive) testing can also increase learning success over months [4], [6], [9], [10], [11]. A randomized study on continuing medical education for postgraduates could not confirm this [12]. However, the studies are not directly comparable with each other due to the large methodological differences.

Immediate feedback on MC questions can increase learning success and reduce frustration [6], [13]. Appropriately, the students complained that some questions were not answered. In the meantime, the seminars have been revised accordingly. A direct comparison of the results of the scattered questions with the summative learning control is unfortunately not possible due to the free text answers in the context of this project and is the content of a future study.

## Conclusion

Overall, there was a high level of acceptance of the digitized seminars; in particular, the challenge posed by formally interspersed questions was rated positively by the students. The seminars will therefore be used in the following semester after revision with regard to the resolution of all questions. 

## Acknowledgements

We would like to thank Ms. Nathalie Petersen, Competence Center Teaching Evaluation, Dean of Studies at the Albert-Ludwigs-University of Freiburg, for her support during the evaluation.

## Competing interests

The authors declare that they have no competing interests. 

## Supplementary Material

List of all digitized seminars. In bold are the seminars with additional contents.
