# Supplementary material for: Interactive, case-based seminars in the digitized pediatrics block internship from the students’ perspective
Source: GMS J Med Educ. 2021 Jan 28;38(1):Doc24. doi: 10.3205/zma001420 (PMC7899105; doi:10.3205/zma001420)
Supplement: List of all digitized seminars. In bold are the seminars with additional contents. [file JME-38-1-24-s-001.pdf]

Attachment 1: List of all digitized seminars. In bold are the seminars with additional contents.

General pediatrics

- **Allergology**
- The child with cough
- The child with swollen knee
- The child with vomiting and diarrhea
- The newborn
- **Endocrinology: Case reports**
- Fever and dysuria
- Child endangerment
- Pediatric Endocrinology
- **Antibiotic therapy in children**
- **U9 in a pediatrician practice**

Neuropediatrics

- The child with impaired consciousness
- The child with headache
- **Normal neurological development**

Hemato-oncology

- The pale child
- The child with a solide tumor
- **Hemostaseology**

Pediatric cardiology

- **Caes reports in pediatric cardiology**
- Cyanosis of the newborn

Attachment 1 to: Heizmann A, Bode S, Forster J, Berger J. *Interactive, case-based seminars in the digitized pediatrics block internship from the students' perspective.* GMS J Med Educ. 2021;38(1):Doc24. DOI: 10.3205/zma001420
